# 20 years of Portuguese drug policy - developments, challenges and the quest for human rights

**DOI:** 10.1186/s13011-021-00394-7

**Published:** 2021-07-17

**Authors:** Ximene RÊGO, Maria João OLIVEIRA, Catarina LAMEIRA, Olga S. CRUZ

**Affiliations:** 1 Research Centre for Justice and Governance (JusGov), University of Minho, Campus de Gualtar, 4710-057 Braga, Portugal; 2 Institute of Sociology of the University of Porto, Via Panorâmica, s/n, 4150-564 Porto, Portugal; 3 Agência Piaget para o Desenvolvimento (APDES) / RECI, Alameda Jean Piaget, n.100, 4001-801 Arcozelo, V.N.Gaia, Portugal; 4 University Institute of Maia (ISMAI), Research Unit in Criminology and Behavioral Sciences (UICCC/ISMAI) and Research Centre for Justice and Governance, University of Minho (JusGov/UM), ISMAI - Avenida Carlos de Oliveira Campos, 4475-690 Maia, Portugal

**Keywords:** Portuguese drug policy, Crime of drug use, Decriminalization, Human rights, Harm reduction, Commissions for the dissuasion of drug addiction

## Abstract

Portugal decriminalized the public and private use, acquisition, and possession of all drugs in 2000; adopting an approach focused on public health rather than public-order priorities. Arguing that the Portuguese Drug Policy Model has not proven influential enough to emancipate drug use from the stigma that associates it either with crime or pathology, this article critically discusses the developments and current challenges the Portuguese drug policy confronts, namely the growing diversity of drug use patterns observed in Portugal as well as in Europe. To this end, international and national legal instruments concerning drugs and official local data were analysed. Despite encouraging results, conclusions indicate that these policies are marked by contradictions and ambiguities that have permeated its history since the very beginning, and modest ambitions, particularly regarding the implementation of harm reduction measures. Moreover, the polemical Supreme Court judgment that reestablished, in 2008, drug use as a crime when the quantities at play exceeded those required for an average individual’s use for 10 days, might have impacted the landscape of drug use penalization. The last decade saw an increase of punitiveness targeted at drug users, including criminal sentences of jail terms. We finish with some suggestions that could be employed in the practical application of drug policy.

## Background

Manichean creeds have long dominated the ideology behind drug policy. Despite the widespread advocacy for evidence-based policies [1] and policies encompassing human rights [2], those creeds conveyed drug use as an exceptionally dangerous behaviour, maintaining till the present day the goal of a drug-free society [3]. Yet, the decriminalization movement seems to be increasingly appealing around the globe [4]. An example of this is the so-called Portuguese Drug Policy Model (PDPM), whose implementation, since 2001, decriminalized the public and private use, acquisition, and possession of *all* illegal drugs (being in this regard quite innovative), as long as they do not exceed the amount required for an average individual’s use for 10 days (Law n. 30/2000, November 29, 2000). The distinction between *soft* and *hard* drugs was abolished [5].

Drug use became an administratively sanctionable misdemeanor, but not a crime, and was placed under the jurisdiction of the Commissions for the Dissuasion of Drug Addiction, created by the Decree-Law n. 130 -A/2001 (January 23, 2001). The PDPM is in line with the belief that the War on Drugs has failed, thus pledging to guarantee greater respect for the rights of people who use drugs; and it is also consonant with the wider European and global trends toward policies that lessen drug use penalties [6].

This paradigm shift, which moved the solution to drug use from the public order to the public health domain - hence differentiating between the user and the dealer, the former seen as an ill person in need of care, and the second as a delinquent - has fueled intense national and international academic debate and stirred antagonistic discourse, sometimes presented as a success [e.g. [7, 8]], sometimes as a failure [e.g. [9]]; and, not surprisingly, substantial international media attention has followed. As Laqueur [10] puts it, the Portuguese decriminalization experience became a kind of *screen onto which drug policy agendas are projected*. Generally speaking, the PDPM is internationally recognized for its humanistic and pragmatic character and as exemplary of a participatory process [11–15], although with varying degrees of consensus [see [16]].

Most analyses about the Portuguese case tend to focus on the encouraging results^1^ regarding drug use prevalence, which stayed reasonably low when compared to other European countries [19], including those that criminalize drug use; the drop of infectious diseases rates, high in 2000 [21], as well the decline of the equally high incarceration rates for drug-related offenses [22, 23]. These tendencies cannot be, however, linearly related with the decriminalization law per se, as Laqueur (10) and Quintas [24] have shown.

Paradoxically, the last decade has seen a sharp increase of criminal sanctions targeted at drug users, including some with jail terms [25–27]. Indeed, prohibitionism has not been discarded. Portugal is a signatory of United Nations Drug Conventions and, despite its efforts to inscribe drug policy within the scope of human rights, it does so within conservative lines, specifically those informed by the right to equal access to health and to non-discriminatory treatment before justice. The debate that frames drug use under the light of the right to privacy and to individual freedom, the right to cognitive freedom; or the right to use drugs, as Van Ree [28] proposed, is peripheral. Similarly, alternative discourses have played an almost marginal role in the Portuguese academic debate; namely those that, recommending a drug-set-setting triad for understanding the phenomenon [29], paved the way for a conception of drug use based on hedonistic motivations, pleasure-seeking, mind-expansion or inner exploration.

Historically contextualizing the emergence of the PDPM, this article critically discusses the major developments and current challenges that Portuguese drug policy confronts in the face of the growing diversity of drug use patterns observed in Portugal. Some of these challenges include 1) the apparent paradox of Portugal having decriminalized the use of drugs and yet registering a sharp increase of punitiveness targeted at drug users over the past decade; 2) the ambiguities and anachronisms that permeate the practices of the Commissions for the Dissuasion of Drug Addiction; 3) and the hesitations regarding the implementation of harm reduction measures, some foreseen in the law since 2001.

To this end, the following documents were analysed: 1) the three main United Nations Drug Conventions texts (1961, 1971, 1988), which constitute the international legal instruments on drugs; 2) the main local legislation on drugs; and 3) the data collected by the General Directorate for Intervention on Addictive Behaviours and Dependencies (SICAD) regarding the drug use situation in Portugal. Three time-frames assume special relevance: 1) between 1970 and 2000, in which the production of local legislation was often pervaded by an ambivalence between punishing or supporting drug users; 2) between 2000 and 2008, in which the crime of drug use disappeared from the Portuguese legal landscape; 3) since 2008, when the crime of drug use has been re-established by the Supreme Court judgment n. 8/2008, for cases in which the amounts identified exceed those established.

The main argument developed is that the PDPM has not proven influential enough to emancipate drug use from the stigma that associates it with either crime or pathology, where it is kept captive. We finish with some suggestions to be applied in practice.

## Oscillating between public order and public health (1970–2000)

Portugal joined the War on Drugs in the seventies, even though drug use was not, at the time, a relevant social problem in the country, nor did the legislator distinguish, until then, between drug use and drug dealing. The utopia of a drug-free society, in Portugal and elsewhere, has been sustained by conservative discourses coined in the sphere of law (political-legal discourses) and in the sphere of health (medical-psychological discourses) which, operating as vehicles of social control, converge in the understanding of drug use as a deviation in relation to the norm, whether in legal (crime) or health terms (pathology).

Meanwhile, the harm reduction movement has challenged global prohibitionism [30] and establishes itself, at least potentially, as a motor of social transformation. The liberation of the drug use phenomenon from the War on Drugs paradigm [31] seems to be at the core of the *double HR* - harm reduction and human rights (see Soares et al. [32]) - in particular in its *strong version*, which fully recognizes the right to use drugs, as opposed to its *weak version*, that advocates mainly health rights [33]. The latter, distant from the activism that originally opposed prohibitionism, unfolds the historic tension between health priorities and profound political change regarding drug use [34], which is also found, as we shall see, in the challenges the PDPM faces.

**Fig. 1:**
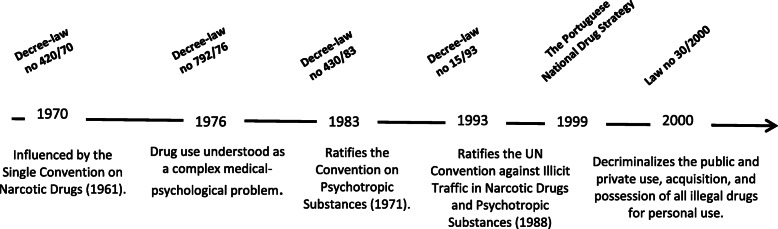
Main Portuguese legal diplomas on drugs. From: Timeline designed by the authors. Cited legal instruments: Decree-law n. 420/70, September 3, 1970. In Diário do Governo n° 204 – I Série. Ministry of Justice; Decree-law n. 792/76, November 5, 1976. In Diário da República n° 259 – I Série. Ministry of Justice; Decree-law n. 430/83, December 13, 1984. In Diário da República n° 285 – I Série. Ministry of Justice and Health; Decree-law n. 15/93, January 22, 1993. In Diário da República n° 18 – I Série. Ministry of Justice; Law n. 30/2000, November 9, 2001. In Diário da República n° 276 – I Série; Decree-law n. 130 -A/2001 (January 23, 2001); The Portuguese National Drug Strategy (1999)

Influenced by the Single Convention on Narcotic Drugs (1961) - well known for establishing the coordinated international fight against drug phenomena - the Portuguese’ Decree-Law n. 420/70 (September 3, 1970) criminalized drug use and regulated the repression of trafficking, but - surprisingly - the latter moderately. At the time, the law emphasized the immorality of drugs and the lack of criminal liability of the user, based upon an exclusively public security perspective to the detriment of health, which was not in line with that convention. Not much later, the Decree-Law n. 792/76 (December 3, 1976) reflected a slight progress: drug use was understood as a complex medical-psychological problem; and, although not providing any response in this area, led to the creation of the *Centre for Drug Study and Prophylaxis* (Fig. 1).

Following the increase in cannabis use and the emergence of heroin - dynamics that took shape after the end of the dictatorship period [7] in 1974 - the Decree-Law n. 430/83 (December 13, 1983) gave the user greater prominence. Change took place under the auspices of the Convention on Psychotropic Substances (1971), which encouraged the treatment and reintegration of users. However, considering drug phenomena as the *plague of our days*, it proposed for trafficking *counter-measures similar to those used against terrorist organizations*, which meant the aggravation of penalties, including provision of new means of obtaining evidence, and the unequivocal criminalization of drug use [35]. The user came to be perceived as captive of either the determinism of crime or of the determinism of pathology [36], in a scheme that Costa [25] calls the *mixed medical-criminal system* - a combination of repressive measures with those of a medical character; resulting in a double stigma that persists, with some variants, to the present day [37].

The Decree-Law n. 15/93 (January 22, 1993) reaffirms the hegemonic medical-penal model, reflecting the rectification of the United Nations Convention against Illicit Traffic in Narcotic Drugs and Psychotropic Substances (1988), which intensifies prohibitionism through a set of proposals, including the criminalization of drug use, which is not, however, mandatory. Still partially in force, it is perhaps the most ambiguous legislation in the Portuguese drug policy panorama. The penal regime of drug use, globally considered, was aggravated [25]: besides the acquisition and the possession for personal use, the use and the cultivation for personal use are also dealt with; jail terms of up to 3 months remained similar to the previous Decree-Law, but where the amounts apprehended exceed those required for 5 days - and here lies much of the novelty - jail time is extended to 1 year. Alongside this, while trafficking remains associated with violent crime, sanctions are lessened, either by slightly reducing prison sentences, or by introducing intermediate categories such as the “less severe trafficking” and the “dealer/user” crimes, both with softer penalties [38].

It is not surprising to find that, although the user was, as early as 1993, *sanctioned by the law almost quasi-symbolically* (Decree-Law n. 15/93), the number of people incarcerated continued to rise and, in 1999, drug-related crimes were the main reason for effective prison sentences, which significantly increased the use of imprisonment without an equivalent increase in crime rates [39]. This tendency was observed until 2003, when - once decriminalization came into force - crimes against property were again in the top spot [39], as they remain nowadays.^2^

Not free from ambivalence, the Portuguese drug policies have made their way from public order to public health domains while, nevertheless, mixing the two concerns. The theme was dealt with as something that disturbs the social order, establishing itself as a commonplace both in the popular imagination and in the political and media agenda. Meanwhile, the medical-psychological model, militating toward abstinence as the sole legitimate therapeutic goal and focusing on the most troublesome side of the phenomenon, found expression in the design of policies that feed the image of the drug user as someone susceptible to compulsive treatment.

Paternalistically, the Decree-Law n. 15/93 states, among its objectives, that the user is *freed from the slavery that dominates him*. The progressive transformation of the status of the user answers old quests, such as the American Psychiatric Association one, which included, in 1934, addictive behaviours in the list of mental illnesses; or the 1971 UN Convention that endorsed treatment and reintegration as an appropriate response instead of punishment. The insistence on pathology rescued drug users from moral condemnation - even if pathologizing undermines the agency and self-determination of people who use drugs [37] - allowing his/her symbolic status to be profoundly transformed; and the phenomenon to finally shift into the public health realm.

At the end of the nineties, the vulnerability of high-risk users raised concerns among the broader society - for example, the HIV epidemic was exploding [12] - and legislative change was imperative. Following Portugal’s National Drug Strategy [40], the transition to a more comprehensive paradigm culminated, in 2000, with the commonly referred decriminalization law.

## The decriminalization law and the Portuguese drug policy model (2000–2008)

The PDPM is inextricable from its socio-historical background. Contrary to what has been observed in other countries - where the discussion about decriminalization has been linked to the increasing prevalence of cannabis use and to a certain normalization of its use among youth [18] - in Portugal, the political debate was driven by the concern about the psychosocial vulnerability of high-risk users, whose long trajectories on drugs have made evident the signs of stigma and social exclusion. Problematic heroin use was the second highest in Europe in 2000 [41] and, although it has been losing its relevance, Portugal remains among the countries with a higher ratio of high-risk opioid use (5,2 per 1000 of the adult population in 2015) [19].

This circumstance engendered a complex challenge - that of devising policies that had a social and human side capable of responding to the situation of exclusion in which drug users found themselves. This might justify the fact that the decriminalization law is based on a dichotomous classification of drug users - dependents and non-dependents - which, already formulated in the Decree-Law n. 15/93, persists to the present day and embodies one of the most relevant anachronisms of the practices of the Commissions for the Dissuasion of Drug Addiction.

With the main goal of widening the social and “sanitary” protection of drug users, which has been quite successful, Portugal decriminalized drug use in 2000. Article 28° of the new Law n. 30/2000, regarding revoked norms, unequivocally states that article 40° (Decree-Law n. 15/93), about the crime of drug use, is derogated (except in what concerns cultivation), as well as other provisions that are incompatible with the present regime.

Among the main outcomes lies the significant increase of the number of drug users who are encompassed by the system, which is not necessarily a surprise: from an average of 1.5 thousand cases per year, between 1993 and 2000, to an average of 4.7 thousand annual cases, between 2001, when the law come to force, and 2014 [34]. This circumstance may reflect the “net-widening effect”, documented in South Australia, following the Cannabis Expiation Notice, in 1987 [42]. Still, it sharply contrasts with what has been observed in California, where a decrease in cannabis detections and referrals was registered after the 1976 Moscone Act, which turned small quantity possession of cannabis into a misdemeanour sanctionable by a fine [43].

But the virtues of the PDPM do not only rely on the decriminalization law per se, but on the set of devices forged and implemented in the meantime. Alongside the legislative changes, it became evident that there was a need to develop more specialized and autonomous coordination mechanisms, namely the Commissions for the Dissuasion of Drug Addiction, responsible for implementing administrative sanctions, and harm reduction structures, lacking at that time.^3^

### Commissions for the dissuasion of drug addiction

The Commissions - under the responsibility of the Ministry of Health - may represent the most groundbreaking feature of the PDPM. By isolating law enforcement from the operationalization of measures outside the criminal arena, the security inclination that so often characterizes drug policies should be obliterated; thus, distinguishing the Portuguese case from others, such as Spain or Italy, which had adopted earlier the administrative approach to sanction drug use [25, 45].

The Commissions’ multidisciplinary teams carry mainly psychosocial intervention, and are accountable for psychological assessment, for providing technical support in determining suspensive measures or sanctioning measures, for referral to health structures and follow-up in the provisional suspension of the procedure, for the determination and execution of those measures, as well as for the application of other alternatives [46]. Police forces are expected to remain the primary source of detection of drug use and subsequent referral.^4^

The Commission’s principles are in line with the hegemonic discourse in what concerns abstinence. Its main goal is *encouraging adherence to treatment, or the decision to abstain from drug use* (Decree-Law n. 130-A/2001). Moreover, while referral to health structures is optional, physically presenting oneself before the Commissions is mandatory for those who are caught using drugs. This circumstance is somehow contradictory to the mainstream perspective, which frames drug use in the health sphere, where consent is pivotal. This is the position, for instance, recommended by the Mental Health Law (Law n. 36/98). The critical issue is that the Commissions - despite being under the Health Ministry’s responsibility, its teams being mainly composed of psychosocial technicians and its goal being prioritizing a health approach - exist with the end of processing administrative offenses and of applying sanctions, a circumstance that somehow constitutes an incurable contradiction.

Divergent from the initial intuition, perhaps fed by the images of drug users in extreme situations of social exclusion, dominant in the end of the twentieth century, it quickly became apparent that the vast majority of the clients of the Commissions were (and are) cannabis users classified as non-dependents. As in previous years, in 2019 the Commissions reported that 90% of all identified drug users - 83% of whom were cannabis users - were classified as non-dependents [27]. The reason behind this circumstance is that cannabis is the most widely used illegal drug [49], in Portugal and elsewhere, its use is socially widespread, and it occurs mostly on the street and in public spaces during leisure time [50]. Overlooking the diversification of drug use patterns, the dependent or non-dependent dichotomy proposed by Law n. 30/2000, and in use by the Commissions, leaves unaddressed who the users targeted by these sanctions are, in terms of their drug use patterns and profiles, and what trajectories led them to this circumstance.

### Harm reduction

Certainly the PDPM facet that has raised the most opposition, harm reduction policies expanded as a direct result of the decriminalization law. Throughout its short history, from clandestinity to political legitimacy, it went through an experimental phase (1993–1998) in which programs (e.g., support offices, street teams and shelters) were developed mostly on the initiative of the civil society rather than by political will [51]. The initial goal was the access to hard-to-reach heroin and crack-cocaine users who resisted the traditional socio-health approaches based on pathology [44]. But the diversification of drug use patterns [22] steered harm reduction to widen its spectrum of intervention to a population different from its original target, underscoring the increasing complexity of current drug use phenomenon and highlighting the necessity of interventions that respond to well-being’ broader claims and that ultimately fall within the human rights sphere.

Two measures of particular relevance have been on standby for nearly 20 years - drug checking services^5^ and drug consumption rooms^6^ - both foreseen in the Decree-Law n. 183/2001 (November 29, 2001), which regulates the national harm reduction policy. Others worth mentioning are the absence of needle and syringe programmes in prison contexts; or the absence of naloxone prescription for outpatient use, either among outreach teams or peers, since it remains exclusive to hospitals and medical emergency services.^7^

Despite the fact that the vast majority of drug users are non-problematic, services are, with some notable exceptions, mainly focused on opioid substitution treatment (partially serving purposes related with public order) and on needle and syringe exchange (with public health preoccupations underneath), thus configuring the *weak version* of harm reduction: the one that advocates mainly health rights, as opposed to its *strong version*, which fully recognizes the right to use drugs [33].

Indeed, and although constituting the main alternative policy to the medical-psychological model of drug-illness and abstinence-centred therapies, harm reduction’s role in pacifying certain territories and its efforts to exert some social control is not to be underestimated [53], reflecting what Roe [34] called the medicalization of political and social problems. The implementation of solely uncontroversial measures has allowed the maintenance of the *status quo*. Despite being humanistic and pragmatic on the ground, political hesitation in this sphere works in compliance with the War on Drugs.

## The crime of drug use and the supreme court of justice 2008′ judgement

In 2008, the Supreme Court of Justice took the position, by judgment (n. 8/2008, August 5), of reestablishing the crime of drug use (article 40°, Decree-Law n. 15/93) when the quantity detected exceeds the average individual use for a period of ten days (behaviour punishable by imprisonment for one year or fine up to 120 days). These quantities are defined by the ordinance Law n. 94/96, March 26, 1996, in use till current days. A number of factors contributed to this situation.

In 2000, and contrary to what has happened with drug use, Law n. 30/2000 kept drug dealing (production, manufacture and trade of illegal drugs) stayed legally framed by the Decree-Law n. 15/93, which penalizes “trafficking and other illicit activities” (article 21°), “less severe trafficking” (article 25°); and the “dealer/user” (article 26°). This last category regards those situations where the individual has the ultimate aim to get substances for personal use that do not exceed the quantities for a medium use of up to five days.^8^

Concerning drug use, article 40° of the same Decree-Law established the crime of drug use and punished it with imprisonment up to three months (or penalty fine up to 30 days); if the quantities exceeded the amount necessary to the medium individual use up to three days, the penalty was of up to one year in jail (or penalty fine up to 120 days). Moreover, the Decree-Law n. 15/93 distinguished between dealing and using, criminalizing both practices, but - as long as this distinction was established - prevented drug use from being legally punished as dealing, regardless of the quantities seized. Differing from what happened with the decriminalization law, no quantitative limit was established for the purpose of distinguishing between the two behaviours.

Law n. 30/2000 sets quantities that shall not be exceeded. However, it does not provide any legal sanctions for those who, as drug users, hold larger amounts. This gave rise to a perturbing possibility: users with less than permitted quantities were sanctioned, even if not criminally; other users, with quantities exceeding those established, would not suffer any sanction, since these sanctions escape the direct provision of the law (regarding the difficulty to categorize certain cases, see Domoslawski [12]).

The Supreme Court of Justice, considering that *it was not intended to legalize drug use*, *but only to decriminalize less severe consumption*, reestablishes the crime of drug use (article 40°, Decree-Law n. 15/93) for cases in which the amounts identified exceed those established. Later, in 2014, the Constitutional Court did not consider the interpretation of the Supreme Court of Justice unconstitutional and validated its position (judgment n. 587/2014, December 3). Although not fully binding, judgement n. 8/2008 is currently used as an uniformizing instrument for judicial decisions. This position has been seen as polemical [24] even among renowned judges, who were persuaded that the Supreme Court action is contrary to the spirit of the decriminalization law [25].

Notably, these sanctions are not directed at dealers, nor at dealers/users, but uniquely to those who have been proven only to be a drug user. It should also be underlined that it is not the quantity that serves to distinguish between use and trafficking. The Portuguese system is a model guided by threshold values only to differentiate between types of use (ones that should be considered a crime or a misdemeanour), and avoiding the hazards associated with the introduction of metrics that, in general, generate more punitive systems [26].

Paradoxically, despite having decriminalized the use of all illegal drugs, Portugal has an increasing number of people criminally sanctioned - some with prison terms - for drug use [25–27]. Regarding criminal sanctions, in 2019, among the convictions under the Drug Law (1883 individuals), drug use (42%) was the second most common, behind drug dealing (58%); no one has been sanctioned for dealing-using [27]. Before 2008, reflecting the decriminalization law, sentences for drug use were almost non-existent and exclusively related to cultivation, which continued to be a crime (article 40° of the Decree-Law n. 15/93, of 22 January).

**Fig. 2:**
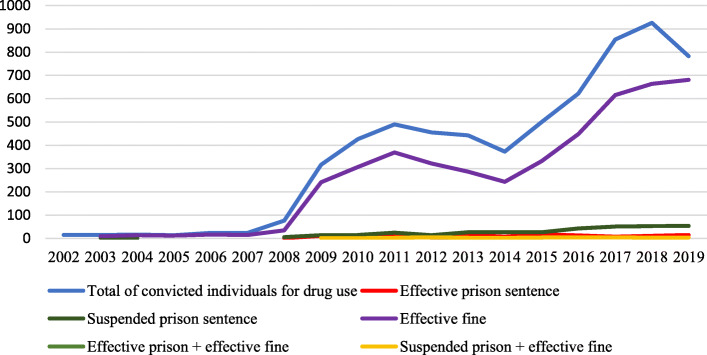
Convicted individuals for drug use, by type of penalty. From: Chart designed by the authors, based on data provided by SICAD – Annexes to national reports on drugs between 2003 and 2020, available at: http://www.sicad.pt/PT/Publicacoes/Paginas/default.aspx

After 2008 (Fig. 2), the sharp increase of sanctions for drug use - which includes fines (suspended or effective), jail time (suspended or effective) and a combination of fines and jail time - is seemingly attributable to the establishment of jurisprudence. According to SICAD [27], in 2019, the Supreme Court judgment n. 8/2008 is explicitly stated in about 99% of the convictions. Moreover, between 2010 and 2019, while convictions for the crime of drug use saw an increase, convictions for drug dealing, including the dealer-user category, registered a decrease [27].

Current data on individuals imprisoned under the Drug Law (December 31, 2019) points to 1862 inmates (the second lowest number in the decade), mainly convicted for dealing (76%), followed by minor dealing (24%). The category ‘other’ represents less than 1% [27]. Remarkably, there is no information available for the crime of drug use. The increase of punitive responses raises the question of what happened in Portugal during the last decade and what is the actual role played by the Supreme Court judgment of 2008.

Administrative sanctions (i.e., operated by the Commissions) and, in particular, trends from 2010 to 2019, also registered a global increase [27]. Among the decisions made on these occurrences (8150 in 2019), predominated the suspensive ones (80%), followed by the punitive (19%) and acquittal (1%). In 2018, compared to previous years, there is evidence of a higher weight of punitive decisions, although the proportion of suspended sentences, punitive sentences and acquittal have remained relatively stable over the past four years [27].^9^ This suggests that, despite suspensive sanctions being the predominant measure used by the Commissions (80%), the rise of punitiveness might not be limited to the criminal sphere.

## Conclusions

Portuguese drug policy has been, since 2000, under great scrutiny, due to the decriminalization approach. Yet, it appears that the PDPM has not proven influential enough to emancipate drug use from the stigma that associates it with either crime or pathology, where it is somehow captive. The analysis carried out allows the conclusion that the PDPM is marked by anachronisms, ambiguity and modest ambition - which reveals the remnants of arguments that see drug use through the lenses of transgression, whether in legal or health terms. These findings lead us to the following final arguments.

### Implications for practice

Since 2009, there has been an increase of criminal sanctions for drug use. A tenacious dilemma - *punishment or support?* - might explain these trends since it is not entirely new. The long-lasting tension between the will to criminalize drug use (and comply with United Nations conventions) and the aspiration to support drug users has been shown since the very beginning by Portuguese legislation. That tension appears to be heightened as the enthusiasm of the decriminalization law faded away and its execution has been eroded by time or routine.

The reestablishment of the crime of drug use - that recaptures drug use in the criminal scope - had blurred the innovative features of the PDPM, standing out, not only as an example of the ambivalence that marks the history of Portuguese drug policy, but perhaps as its most serious setback, putting drug users at risk of criminal sanctions. Since the reestablishment of the crime of drug use is the sole responsibility of the legislator, it appears that the Supreme Court of Justice did more than interpret the law, surpassing its sphere of competence, an understanding that has now the “seal of guarantee” of the Constitutional Court.

In this sense, it seems that the legislator - keeping, first, the spirit of the decriminalization law and, second, adopting an evidence-based orientation - should return proven cases of drug use to the Commissions, the body under which this behaviour was initially placed, assuring that drug use stays, as originally, an administratively sanctionable misdemeanor, but not a crime. Adequate and tailored sanctions for these cases, that might foresee an update of the *old* ordinance Law n. 94/96, the table that regulates the quantities permitted, could be needed.

A second important implication for practice is related to the Commissions’ undertakings. Notwithstanding the fact that Commissions might constitute the most original feature of the PDPM, the drug use landscape is marked by an ever-increasing heterogeneity of drug use patterns [22] for which the binary categorization employed, that of dependent and non-dependent users, is inappropriate. More finely graded drug use pattern classifications are needed in order to accommodate and better address past decades’ sociocultural transformation. These transformations include the progressive substitution of compulsive consumption behaviours that are being replaced by self-care [55] and the diversification and multiplicity of drug uses [56], in which non-problematic ones are the vast majority, and in which hedonistic motivations assume a central role [57].

Furthermore, considering that consent and self-determination are crucial aspects of any intervention in the health realm, we endorse that appearance before the Commissions, instead of being mandatory, should be conditional on the consent of the individual, even if administrative sanctions are still to apply. The decision to undergo any type of diagnosis, clinical evaluation or therapeutic intervention is up to the citizen, within the scope of their rights, freedoms, and guarantees - as is recognized by, for example, the Law of Mental Health. Were it otherwise, it would create a regime of exception within the scope of the response to drug use.^10^

Thirdly, regarding harm reduction policies, it might be observed that these are somehow still oscillating between the concerns that make justice and health priorities. Harm reduction measures that fall strictly within the health scope - or whose absence would pose obvious challenges in terms of public health and/or social order - tend to face little or no opposition. This mirrors how the notion of drug use as something in the pathological scope is well assimilated by Portuguese society and political affairs. Controversy and political opposition, and perhaps social reluctance, seem to arise when harm reduction policies go beyond pathology and highlight issues related to the well-being and the agency of the drug user.

Good examples of measures which provoke such pushback (whose implementation we nevertheless highly recommend) include, for instance, the resistance to implementing drug checking services, crucial to assisting informed choices on drug use. Or the failure to provide naloxone outside medical settings, which, though addressing pathology, would imply transferring powers from the hands of doctors to the hands of the individual. Other recommended programmes in need of implementation include needle and syringe programmes in prison contexts and, in line with the sociocultural transformation of drug use, tailored programmes aimed at poly-drug use, the most common consumption pattern [58]; and gender-sensitive programs, which are virtually nonexistent.

### The quest for human rights

Following Portuguese sociocultural transformation and the diversification of drug use patterns, observed later in Portugal, but somehow identical to main European trends, drug use is escaping its label as something that unfolds at the margins of society. Besides the focus on the normalization of the use of certain illegal drugs and on recreational uses [59], research has addressed patterns of use that are defined as functional [60], non-dependent [61], religious [62], healthy [63], socially-integrated [64] and non-problematic [57, 65]. These data gather momentum to advocate for the need to consolidate the on-going paradigm shift, namely by strengthening the notions of health that are broad enough to consider well-being and not solely pathology, which obviously is a poor concept when it comes to empowering and respecting the dignity of people who use drugs. As mentioned, such pathologizing serves to undermine the agency and self-determination of people who use drugs [37].

Yet, the most ambitious challenge is to promote a debate that places the drug use phenomena in the Human Rights realm, thus favouring the respect for the principles of individual freedom and the right to an informed choice. The interplay between drug phenomena and human rights is far from linear (see Bone [2]). The core conflict has remained quite immutable since the beginning of global prohibitionism: the interest of States in restricting the access and use of certain substances and the interest of the individuals in using them, which unfolds a parallel conflict on a different, but not distant, level: between the health of the population or sovereignty over one’s body - which is to be prioritized? Different perspectives take shape in the debate, from conservative trenches (as the right to equal access to health and justice) to the right to use drugs for certain purposes - namely ritual-religious ones, these contemplated in the UN Conventions - or simply the right to use drugs [66]. The more liberal movements advocate the need to “Legalize it All”, displaying a diversity of arguments that vary among the right to privacy and to individual freedom (which can be both read in the light of article 12° of the Universal Declaration of Human Rights) or to the cognitive freedom (article 18°); and work around notions of informed choice and individual sovereignty over the body [33].

In line with the United Nation General Assembly Special Session Against Corruption [67] - which emphasizes a comprehensive approach, but delimits it within the framework of the human rights of health and justice - the debate about the right to use drugs is nearly absent in the Portuguese political, social and academic panorama. On the contrary, and despite the seemingly innovative character of Portuguese drug policy, there have been numerous hesitations, namely with regard to the regulation of medical cannabis, which was approved by the Portuguese government in 2018 (Law n. 33/2018, July 18), and which itself can be seen as a matter of human rights [67]. One of the difficulties lay in some political parties’ claim for a clear distinction between the medical and recreational use, something well challenged elsewhere [66, 68]. Meanwhile, three proposals (one in 2015, two in 2019) regarding the regulation of recreational use of cannabis took place in the Portuguese parliament, most of them triggered by civil society organizations, and all unsuccessful.

These hesitations do not match the progressive transformation of drug use patterns, local and global, nor the several challenges, coming from different quadrants, posed to the War on Drug*s*, within what some call the *transformational movement* [46]. Opposition to punitive drug policies keeps growing [30]. Finally, casting drugs as *malevolent agents that* allow *classifying users as bad or sick (or both)* [31] became a fabrication that eases the *stigmatization* of users and *human rights violations* [31]. Furthermore, it can be argued that in Portugal, as in other countries with a prohibitionist approach, there are ‘victims’ originated, not necessarily by drug use in itself, but by drug laws [66] - as it is the case for the individuals that are being convicted by the crime of drug use, some of them with effective prison sentences - which, again, can be seen as a harm to human rights [67].

A non-paternalistic mid-term view, that broadens the scope of public health to include, for instance, the right to an informed choice and the right to risk taking behaviour - despite the controversial degree of control that individuals might exert over their bodies [33] - might offer a possibility. The opportunity is taking shape. It is expected that the regulation of recreational use of cannabis will be soon brought again to public debate. Yet, to keep no distinction between *soft* and *hard* drugs - as Portugal’s National Drug Strategy [40] has proposed and Law n. 30/2000 tried to materialize - and to fully accomplish the protection of individual freedom in the light of human rights, one should think about regulating all drugs.

Attributable, at least partially, to the many anachronisms, ambiguities and hesitations described above and, specially, to the reestablishment of the crime of drug use, the last decade has seen a clear increase of punitiveness targeted at drug users, which is not, we believe, in line with the decriminalization law neither with the set of devices forged and implemented alongside the legislative changes. Awareness regarding these challenges is worth further research, in particular, if the innovative spirit of Portugal’s National Drug Strategy [40] is to be kept alive.

## Data Availability

In references.

## Abbreviations

SICAD: General directorate for intervention on addictive behaviours and dependencies
PDPM: Portuguese drug policy model

## Footnotes

1 Positive results include a reduction in mortality and infections associated with dependent behaviour and, among youth, a reduction in the prevalence of recent cannabis use, a postponement of the age of initiation and an increase in perceptions of related-risks [17]. Notwithstanding some fewer encouraging results, especially those related to the general population (aged 15 to 74 years), whose increase in prevalence and intensity of cannabis use [17] may reflect the normalization thesis [18]. Overall, one might observe a reasonably low level of drug use when compared with most European countries [19], which contrasts to what was witnessed at the end of the last century, a period marked by social and political alarm around drug phenomena. Consonant, social representations are now quite distinct from those that dominated the country earlier and which understood drug use as a serious social problem [e.g., [20]].

2 Note that the decriminalization law has no impact on incarceration for drug trafficking, since this behavior is regulated by Decree-Law n. 15/93, still in force. These tendencies might indicate either a change in law enforcement priorities, since law enforcement enjoys a certain discretionary power [38]; different sentencing practices [10, 26] or a combination of both.

3 In 2007, the Institute on Drugs and Drug Addiction won the status of Public Institute. This decision was contradicted 4 years later, in 2011, when the institute was extinguished and SICAD was established in its place, which meant that the operationalization of interventions in drug behaviours was delegated to the Regional Health Administrations [44]. This change, based on criteria of efficiency and administrative rationality, reinforced intervention on drugs in the health realm, giving less emphasis to a social and human rights approach.

4 Spain and Italy show crucial differences when compared to Portugal. In Spain, use or possession for use, when in a public space, constitutes a serious infraction, framed in the *Ley de Seguridad Ciudadana* (i.e., law on citizen security), and is sanctioned by internal security authorities [25]. This circumstance does not exclude the fact that the normalization of cannabis use is more vigorous in Spain [47] - where *Cannabis Social Clubs* were formed - than in Portugal, a trend that has not been followed by political and legal normalization, since the Supreme Court of Justice has sentenced elements of those clubs with prison sentences, even if lightweight [38]. In Italy, drug use sanctions - of essentially a security nature - fall within the competence of the *prefetto* [48].

5 There are two exceptions to mention: *Check-!n Free Mind* and *Kosmicare,* services implemented by non-governmental organizations, have occasional interventions at festivals and parties, and *Kosmicare* has operated in Lisbon two days a week, since November 2019.

6 Exceptions made for one Drug Consumption Room operating since May 2021, and one mobile unit operating since April 2019, both in Lisbon.

7 In 2007, a Needle and Syringe pilot-project ran in the Portuguese prisons for 6 months, but was not used by a single prisoner, see Sander et al. [52]. In January 2020, SICAD started a pilot-project for naloxone distribution among outreach teams in Lisbon and, in April, among outreach teams in the North of Portugal. In May 2021, SICAD made available naloxone for 6 months to all outreach teams.

8 Even though much of the “small” dealing is to sustain drug use, it turns out that the legislator requires - to punish the agent as a dealer-user - that he/she practices the behaviours referred in article 21° with the exclusive purpose of personal use, which immediately precludes the punishment of the agent as such [35, 54].

9 As in previously years, in 2019, the vast majority (94%) of the occurrences involved only one drug: 83% only cannabis, 7% only cocaine, 4% only heroin and about 1% any other drug (mainly ecstasy) [27]. Regarding the patterns of drug use of the individuals involved in these administrative sanctions, the majority is classified as non-dependent users (90%).

10 When a person required to appear before the Commissions fails, in fact, to appear, it is common practice to apply the most minor of sanctions, depending on the substance in question.
